# Primer-Treated Ceramic Bracket Increases Shear Bond Strength on Dental Zirconia Surface

**DOI:** 10.3390/ma13184106

**Published:** 2020-09-16

**Authors:** Ga-Youn Ju, Bum-Soon Lim, Wonjoon Moon, Shin-Young Park, Soram Oh, Shin Hye Chung

**Affiliations:** 1 Department of Dental Biomaterials Science, School of Dentistry, Seoul National University, Seoul 03080, Korea; espoir840126@naver.com (G.-Y.J.); w.moon@snu.ac.kr (W.M.); 2 Department of Dental Biomaterials Science, School of Dentistry and Dental Research Institute, Seoul National University, Seoul 03080, Korea; nowick@snu.ac.kr; 3 Department of Dental Science, School of Dentistry and Dental Research Institute, Seoul National University, Seoul 03080, Korea; nalby99@snu.ac.kr; 4 Department of Conservative Dentistry, School of Dentistry, Kyung Hee University, Seoul 02447, Korea; soram0123@gmail.com

**Keywords:** ceramic bracket, shear bond strength, bonding, phosphate-based primer, dental zirconia

## Abstract

The purpose of this study is to evaluate the shear bond strength (SBS) of a primer-treated ceramic bracket on dental zirconia and to compare it with conventional ceramic bracket bonding on surface-treated zirconia. Sintered and finished dental zirconia was sandblasted. Samples were divided according to the treated surfaces: no treatment (X), ceramic primer on zirconia (Z), ceramic primer on bracket base (B), and ceramic primer on both zirconia and bracket base (ZB). The ceramic bracket was bonded on zirconia and SBS was measured before (T_0_) and after 10,000 cycles of thermocycling (T_f_). The failed surfaces were examined under field emission scanning electron microscope (FE-SEM), and adhesive remnant index (ARI) was evaluated. SBS was significantly higher in ZB and significantly lower in X in both T_0_ and T_f_. There was no significant difference between Z and B. In X and B, adhesive failure occurred while ZB showed mixed failures. There was no apparent change in the zirconia surface except for the existence of some adhesive and resin remnants. The application of ceramic primer on the bracket base increased the bonding strength to the level of conventional bonding with fewer adhesive remnants. The highest bonding strength was obtained when the primer-treated bracket was bonded on the primer-treated zirconia.

## 1. Introduction

Increasing interest in esthetics and the need to overcome the disadvantages of traditional porcelain-fused metal crowns, e.g., the low strength of porcelain and greyish shade of gingiva from metal substructures, have resulted in the development of a wide range of ceramic systems [1,2]. Among them, the use of dental zirconia has increased with the development of computer-aided manufacturing (CAM) devices. 

Polycrystalline zirconia, a frequently used ceramic system in load bearing areas with esthetic demands, primarily consists of yttria-stabilized tetragonal zirconia polycrystals (3Y-TZP) [3,4,5]. The phase transformation of Y-TZP from tetragonal to monoclinic under stress conditions increases the particle volume and inhibits crack propagation, resulting in high flexural strength [3,4,6]. Due to its outstanding mechanical properties, zirconia has been used in inlays, onlays, crowns, post-and-core systems and as frameworks for porcelain fused zirconia (PFZ) restorations [4,7]. Although PFZs provide enhanced esthetics, chipping and delamination of the veneered porcelain limit their use in dental restoration [4,5,8]. With the development of zirconia of increased translucency, the use of full-contoured zirconia (FCZ) is increasing for both anterior and posterior restorations [5].

Zirconia is acid-resistant because it does not contain silica particles, unlike most other dental ceramics. This makes it less reactive to hydrofluoric acid etching, an effective surface treatment method for other glass ceramic systems [2,5,9,10,11,12,13]. It has been suggested that air particle abrasion or tribochemical silica coating can enhance the resin bond strength on zirconia [9,10,11,12]. However, in the latter case, the attachment of silica on the zirconia surface is not predictable after aging processes [9]. In zirconia restorations, such as inlays and crowns, the application of primers or bonding systems with functional monomers (e.g., 10-methacryloyloxydecyl dihydrogen phosphate; 10-MDP) on the roughened surface is recommended to increase the bonding strength [9,12]. 

As the number of adult patients going through orthodontic treatment is growing, the number of cases of bracket bondings on zirconia restorations are also increasing. The problem of bracket bonding failure on posterior teeth can be solved by using other devices such as orthodontic bands. However, in the anteriors, where esthetic considerations are important, there is a frequent need of minor tooth movement due to traumas that cause tooth loss or fracture. In these cases, bracket bonding to zirconia is often inevitable for forced eruption in a short period. Previously, primer-treated metal brackets were used to increase the bond strength on gold alloy surfaces [14,15], but there is a lack of studies about the bonding of primer-treated ceramic brackets on high translucency FCZs. 

Different from previous work that focused mostly on surface treatment of zirconia, in this study, the shear bond strength (SBS) of primer-treated ceramic brackets was tested and compared with those treated by conventional bonding protocols, before and after artificial aging. This study shows that the new strategy, i.e., primer treatment on the bracket base, can not only sufficiently increase the SBS of brackets, but can also provide an option for stronger bonding on zirconia.

## 2. Materials and Methods 

### 2.1. Zirconia Specimen and Bracket Bonding

Eighty high translucency zirconia (LAVA Plus, 3M ESPE, St. Paul, MN, USA) specimens of 15 (width) × 15 (height) × 2 (thickness) mm were prepared and sintered according to the manufacturer’s instructions. Each specimen was finished with a diamond disc (MD-Piano, Struers, Ballerup, Denmark) up to 500 grit under water irrigation to obtain uniform surface roughness. The samples were cleaned in an ultrasonic bath with distilled water for 10 min, and then dried. The surfaces of the samples were sandblasted with 50 μm alumina at a vertical distance of 20 mm for 20 s in a circular motion under a pressure of 0.4 MPa. 

**Figure 1:**
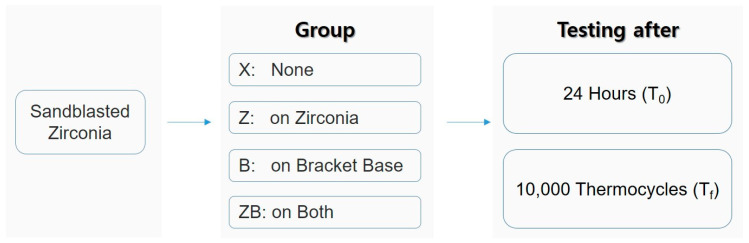
Flowchart of the experiment.

The zirconia samples were randomly divided into four groups (*n* = 20) (Figure 1). In X, no surface treatment was done on the alumina blasted zirconia surface nor on the bracket. In Z, a ceramic primer (Clearfil ceramic primer, Kuraray, Tokyo, Japan) was applied on the zirconia surface. In B, a ceramic primer was applied on the bracket base. In ZB, a ceramic primer was applied on both the zirconia surface and the bracket base.

In Z, B and ZB, after the surfaces were primed, an adhesive primer (Transbond XT adhesive primer, 3M Unitek, Monrovia, CA, USA) was applied on the zirconia surface. Then, a monocrystalline ceramic bracket with a spherical attachment on the base (Perfect Clear II, Hubit, Uiwang, Korea) was bonded using adhesive paste (Transbond XT adhesive paste, 3M Unitek, Monrovia, CA, USA) under gentle pressure. After removing the excess paste, the brackets were light-cured (Elipar Free Light 2, 3M ESPE, St. Paul, MN, USA) for 10 s on each side for a total of 40 s (Table 1).

### 2.2. Surface Evaluation

The surface of the bracket base (as-received) was observed under field emission scanning electron microscopy (FE-SEM; S-4700, Hitachi, Tokyo, Japan). The retentive structures of the intact bracket base were analyzed with an energy dispersive x-ray spectrometer (EDS; EMAX 7200H, HORIBA, Stanmore, UK). The surface roughness ($R_{a}$) of the finished and sandblasted zirconia surfaces was determined using a confocal laser scanning microscope (CLSM; LSM 800-MAT, Carl Zeiss MicroImaging GmbH, Jena, Germany). Each surface was measured three times. 

### 2.3. Shear Bond Testing and Failed Surface Observations

After the bracket bonding, the samples were stored in 100% relative humidity at 37 °C for 24 h. Half of the randomly distributed samples were tested after 24 h of storage. The other half underwent an aging process using a thermal circulator (DTRC-640, Jeiotech, Daejeon, Korea) for 10,000 cycles at 5 °C and 55 °C. The dwelling time was 30 s and the transfer time was 20 s. SBS was measured with a universal testing machine (Lloyd LF Plus, Lloyd Instruments Ltd., Fareham, UK) at a crosshead speed of 0.5 mm/min. The bracket-zirconia specimens were embedded in acrylic molds, and were then fixed on a jig. The crosshead was aligned as closely as possible to the zirconia surface so that it could exert a vertical shear force upon the brackets. Also, the center point of the crosshead was set to meet at the center point of brackets for equal force distribution. The SBS at failure was calculated in MPa by dividing the maximum load (N) by the area of the bracket base (12.24 mm^2^). The bond failure interface was evaluated under FE-SEM, and both failure mode and ARI (A standardized index that demonstrates the amount of remnant in the bonding interface after bonding failure) were evaluated using modified ARI scores (Table 2) [16]. 

### 2.4. Statistical Analysis

Surface roughness before and after sandblasting was analyzed by Mann-Whitney test. Shear bond strengths among the experimental groups were analyzed by Kruskal-Wallis test, since the data were proven to be nonparametric. Shear bond strengths between T_0_ and T_f_ were analyzed by Student’s *t*-test, since the data were parametric and homogeneous. SPSS was used for the statistical analysis (version 22.0; IBM Corp., Armonk, NY, USA). A *p*-value lower than 0.05 was considered statistically significant.

## 3. Results

### 3.1. Microstructural Observations

**Figure 2:**
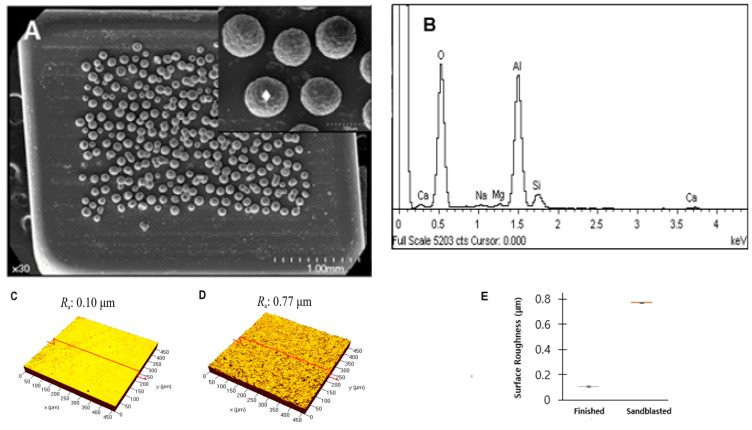
SEM images, EDS analysis and roughness. (**A**) SEM images of the bracket base at ×30 and ×300. (**B**) EDS analysis of the spherical retentive microstructures (white diamond in A). (**C**) CLSM images of the zirconia surface before (median surface roughness (R_a_): 0.104 μm) and (**D**) after the sandblasting (median surface roughness (R_a_): 0.773 μm). Sandblasted surface (**D**) showed evenly distributed microroughness. (**E**) Surface roughness (R_a_) before and after sandblasting.

The surface finished to 500 grit with a diamond disc showed shallow scratch lines without directionalities with a median surface roughness (R_a_) of 0.104 μm. Sandblasted surfaces showed multiple short but deeper scratches that were uniformly distributed with increased median surface roughness (R_a_) of 0.773 μm (Figure 1). The bracket base showed multiple spherical-shaped retentive beads (diameter of approximately 80 to 100 μm) which were only attached to the center part excluding the outer margins (approximately 800 μm in width). The sphere-shaped retentive beads showed characteristic microrough surfaces, and the EDS evaluation revealed their compositions (Figure 2).

### 3.2. Shear Bond Testing and Failed Surface Observations

**Figure 3:**
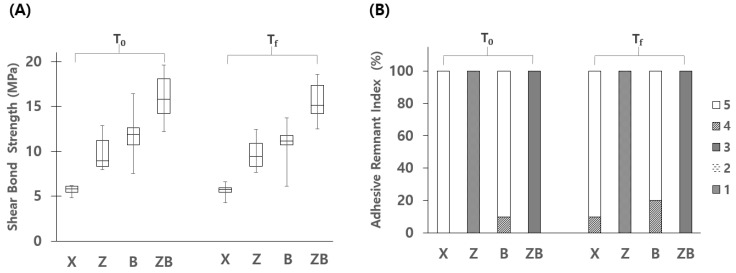
Results of SBS and ARI. (**A**) Comparison of SBSs at T_0_ and T_f_. ZB showed the highest while X showed the lowest. (**B**) Comparison of modified adhesive remnant index (ARI) scores before (T_0_) and after (T_f_) the thermocycling.

Without thermocycling (T_0_), significantly higher bonding strength was obtained in ZB, whereas it was significantly lower in X. Z and B showed no significant differences between the groups (*p* > 0.05). After thermocycling (T_f_), SBS significantly decreased in X (*p* = 0.000), B (*p* = 0.007), and ZB (*p* = 0.001), while there was no significance found in Z (*p* > 0.05). Still, ZB which was treated with ceramic primer on both the zirconia surface and the bracket base showed the highest SBS, while X showed the lowest (Figure 3A).

In ARI scoring, the small amounts of tagged resin which remained between the retention beads of the bracket were not considered. In T_0_, all the samples of X showed adhesive failure at the zirconia and adhesive resin interface with no resin was left on the zirconia surface (ARI score 5). All the samples of Z showed adhesive failure at the resin and bracket interface with the resin left on the zirconia surface (ARI score 1). In B, most of the samples showed adhesive failure at the zirconia and resin interface with no resin left on the zirconia surface (ARI score 5). All the samples in ZB showed mixed failure with some resin left on the zirconia surface as well as on the bracket base (ARI score 3).

**Figure 4:**
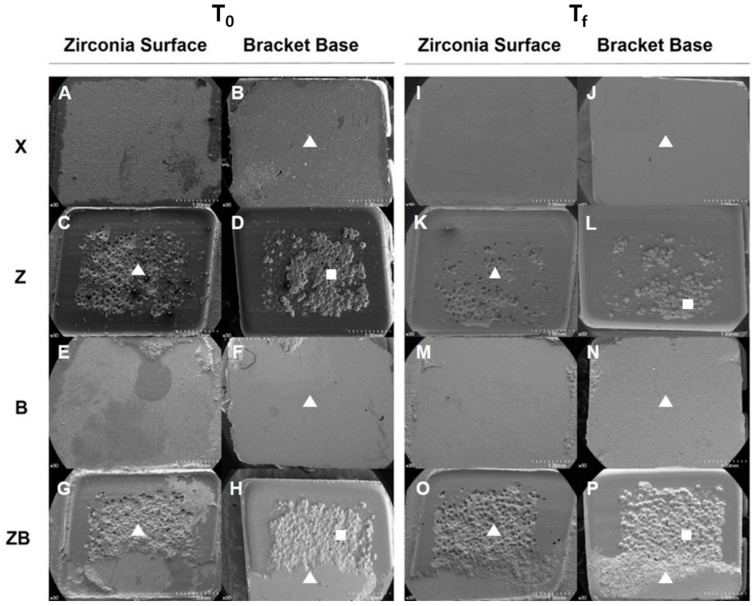
Representative SEM images of bonding interface (×30). Adhesive failure surfaces of zirconia (**A**,**C**,**E**,**G**) and bracket (**B**,**D**,**F**,**H**) in case of T_0_. Adhesive failure surfaces of zirconia (**I**,**K**,**M**,**O**) and bracket (**J**,**L**,**N**,**P**) in case of T_f_. triangle (resin); square (captured resin between the retention beads). (**A**,**E**,**I**,**M**) showed adhesive failure at the zirconia-resin interface; (**C**,**K**) showed adhesive failure at the resin-bracket interface; (**G**,**O**) showed mixed failure at the zirconia-resin-bracket interface.

In T_f_, 90% of the samples of X showed adhesive failure at the zirconia and resin interface. In Z, all samples showed adhesive failure at the resin and bracket interface, in which remnants of resin were left on zirconia surfaces (ARI score 1). In B, adhesive failures with no resin remaining on the zirconia surface mainly occurred (ARI score 5). Most of the samples in ZB showed mixed failure in which some resin was left on the zirconia surface as well as on the bracket base (ARI score 3) (Figure 3B and Figure 4).

## 4. Discussion

This study evaluated the effect of the ceramic primer treatment on the SBS between zirconia and the bracket. All the zirconia surfaces were uniformly prepared to obtain the surface roughness of full-contoured zirconia crown prepared by diamond cutting burs. Micromechanical retention formed by sandblasting is a simple and effective way to increase the bonding strength, despite the risk of decreased mechanical properties of the zirconia [17,18]. In this study, the conditions of sandblasting, i.e., its time, particle size, pressure, movement and distance, were determined to attain the maximum bonding strength within a range not compromising the physical properties of zirconia. The micro-undercuts and roughness of the zirconia were also increased by sandblasting, but its effect on bonding was limited. Furthermore, decreased bonding strength after the thermal aging process, as previously reported, was observed [19].

When the surface was sandblasted only (group X), the lowest bonding strength was obtained, which decreased by more than 64% after aging. Thermocycling is an artificial aging method to simulate the long-term use of the brackets in situ [20,21]. It can also evaluate the hydrolysis resistance of the bonding interface [22]. Performing 10,000 cycles of thermocycling has a similar effect to approximately 12 months of use in the oral cavity [23]. When the bonding interface was exposed to the oral cavity, a significant decrease in bond strength was reported after thermocycling [24,25]. Considering that the bonding strength required by natural teeth is around 6–8 MPa, it could be assumed that sandblasting without primer treatment would yield clinically insufficient bonding strength [26,27].

In the cementation of zirconia restoration with less retention, the application of 10-MDP in combination with sandblasting of the inner surface is recommended for effective long-term bonding [28]. It is known that chemical bonding of the primer is achieved between the functional monomer, such as 10-MDP, and the metal ions of the ceramic surface [29,30,31,32]. From the result of this study, the bonding strength was significantly increased after the primer treatment (Z, B and ZB), regardless of its groups, so it could be suggested that chemical bonding could have indeed occurred. Also, sufficient bonding strength was shown even after the thermocycling.

The major components of the spherical retentive beads on the surface of the bracket base were aluminum and oxygen. Alumina also forms a chemical bond with the 10-MDP because it is a metal oxide like zirconia [29]. In B and ZB, on which bracket bases were treated with the primer, the possibility of chemical bond formation between the bracket and the primer could have contributed to the increased bonding strength.

A significantly higher bonding strength was observed when the primer was applied to both the zirconia and bracket surfaces (ZB), even after the aging process. It seemed that the 10-MDP containing primer clearly played a role in increasing the bonding strength between zirconia/resin and also between the ceramic bracket/resin. Although the ceramic primer also contained 3-methacryloxypropyltriethoxysilane, its effect on bonding strength was not considered in this study, since the effect of silane is known to be weakened in acidic environments due to self-condensation [33]. Also, it was discovered that improved crosslinking capacity of the siloxane network via intermolecular reactions could stabilize 10-MDP [34].

When the primer was applied only to the ceramic bracket base (B), the bonding strength increased to as much as that of primer-treated zirconia (Z). From the results of ARI, 100% of the samples in Z showed that the resin had continuity with the zirconia surface, meaning that the debonding stress was concentrated at the resin/bracket interface. In 80–90% of the samples in B, on the other hand, the bonding strength of resin/bracket exceeded that of zirconia/resin, so there was no adhesive resin left on the zirconia surface. This phenomenon suggests its potential for clinical applications, considering that proper bracket removal is also important in orthodontic treatments. As such, being supported by ARI, a better zirconia surface cleanness was obtained when a surface-primed bracket was removed under shear stress. This implies that fewer remnants on zirconia would decrease the chair-time on the day of bracket removal.

There has been considerable research on the use of MDP-containing primers for zirconia bonding. Many previous works have successfully showed its prominent contribution in increasing SBS of metal brackets on zirconia [35,36]. However, they usually did not consider ceramic brackets nor their various bonding strategies. Since esthetic considerations during orthodontic treatment are essential these days, investigations of the relationships among ceramic brackets, the MDP-containing primer and zirconia, as presented in this work, will have even more clinical implications in the future.

Despite the newly discovered advantages of the study, it also had some limitations. Adhesion in the oral environment is a very sophisticated process and is vulnerable to many factors. For example, pH changes of saliva and the presence of bacteria or chemical byproducts generated by digestion, respiration, etc. might affect the SBS. However, the experimental conditions in the study were limited to the most optimal situation. Further studies conducted under situations better simulating biological and chemical interactions of the oral cavity would definitely extend the scope of the present experiments.

Moreover, the zirconia specimens prepared in this study were not exactly representative of clinical situations. The specimens were finished with a diamond disc up to 500 grit. This was successful in providing a clean bonding surface, but it could not completely simulate the final roughness of zirconia in the clinic. Also, when preparing zirconia specimens, alumina blasting pressure could have induced phase transformation of zirconia from tetragonal to monoclinic. It was reported that a pressure of 0.23 MPa resulted in a 7% phase transformation [37]. In this study, 0.4 MPa, an even higher pressure, was used. Thus, it is highly probable that the experimental results were affected by phase transformation in the zirconia. The relationship among high blasting pressure, phase transformation, and their clinical acceptability should be tested further in future studies.

To further evaluate the significance of this study, the surface quality before and after bonding could have been observed. Based on that result, it may be possible to determine whether the new bonding strategy suggested here induced surface damage during debonding.

## 5. Conclusions

From this study, it was concluded that primer application on the bracket base increases bonding strength to a sufficient level with less adhesive paste on the zirconia. In addition, the highest bonding strength was achieved by applying ceramic primer on both the bracket base and zirconia surfaces. Based on these results, dentists would be able to apply various bonding strategies depending on the situation, e.g., primer on the bracket for quick bonding and primer on both the bracket and the zirconia for strong bonding.

## Figures and Tables

**Table 1:** Materials used in this study.

Brand Name	Composition	LOT No.	Manufacturer
LAVA Plus high translucency zirconia	Tetragonal polycrystalline zirconia, 3mol-% Yttria, Alumina	515920	3M ESPE, USA
Clearfil ceramic primer	3-Methacryloxypropyl triethoxy silane, * 10-MDP, Ethanol	240010	Kuraray, Japan
Transbond XT adhesive primer	* TEGDMA, Bisphenol A diglycidyl ether dimethacrylate, Hydroquinone, Camphorquinone, Triphenylantimony, 4-(Dimethylamino)-Benzene ethanol,	ER7BS	3M Unitek, USA
Transbond XT adhesive paste	Bisphenol A diglycidyl ether dimethacrylate, Bisphenol A Bis(2-hydroxyethyl ether) dimethacrylate, Silane treated quartz, Silane treated silica	ER7BS	3M Unitek, USA

* 10-MDP (10-methacryloyloxydecyl dihydrogen phosphate). TEGDMA (Triethylene glycol dimethacrylate).

**Table 2:** Criteria of modified adhesive remnant index (ARI) scores.

Score	Criteria
1	Entire composite remained on zirconia
2	More than 90% of the composite remained on zirconia
3	More than 10% but less than 90% of the composite remained on zirconia
4	Less than 10% of the composite remained on zirconia
5	No composite remained on zirconia
